# Combined association of aerobic and muscle strengthening activity with mortality in individuals with hypertension

**DOI:** 10.1038/s41440-024-01788-3

**Published:** 2024-08-13

**Authors:** Younghwan Choi, Duck-chul Lee, Yunmin Han, Hoyong Sung, Jiyeon Yoon, Yeon Soo Kim

**Affiliations:** 1 Department of Physical Education, College of Education, Seoul National University, Seoul, South Korea; 2 School of Education, University of Pittsburgh, Pittsburgh, PA USA; 3 Department of Physical Education, Korea Military Academy, Seoul, South Korea; 4 Department of Kinesiology, Iowa State University, Ames, IA USA; 5 Institute of Sport Science, Seoul National University, Seoul, South Korea

**Keywords:** Hypertension, Mortality, Muscle-strengthening activity, Physical activity guidelines

## Abstract

Evidence on the association between meeting both aerobic physical activity (PA) and muscle-strengthening activity (MSA) guidelines with mortality in individuals with hypertension is scarce. We included 34,990 adults from the 2007 to 2013 Korea National Health and Nutrition Examination Survey, linking mortality follow-up data until 2019. Adherence to PA guidelines was assessed based on the current PA guidelines using a self-reported questionnaire and categorized as follows: meeting MSA only, aerobic PA only, both MSA and aerobic PA, or neither. Associations of hypertension and adherence to PA guidelines with all-cause and cardiovascular disease (CVD) mortality were examined using Cox proportional hazard models. Over 9.2 years, 1948 participants died from any cause and 419 from CVD. Meeting both PA guidelines was associated with the lowest risk of all-cause and CVD mortalities in the total sample regardless of hypertension status. In individuals with hypertension, meeting aerobic PA guidelines only had a 24% lower risk of both all-cause and CVD mortality, and meeting both PA guidelines further reduced risks by 40% and 43%, respectively; however, meeting MSA guidelines only was not associated with either all-cause or CVD mortality. In individuals without hypertension, only meeting both MSA and aerobic PA guidelines, but not meeting either MSA or aerobic PA guidelines, showed reduced risk of CVD mortality. In Korean population, non-hypertensive individuals who met both guidelines had a lower risk of CVD mortality. However, hypertensive individuals showed a reduced risk of both all-cause and CVD mortality when meeting aerobic PA or both guidelines, but not MSA alone.

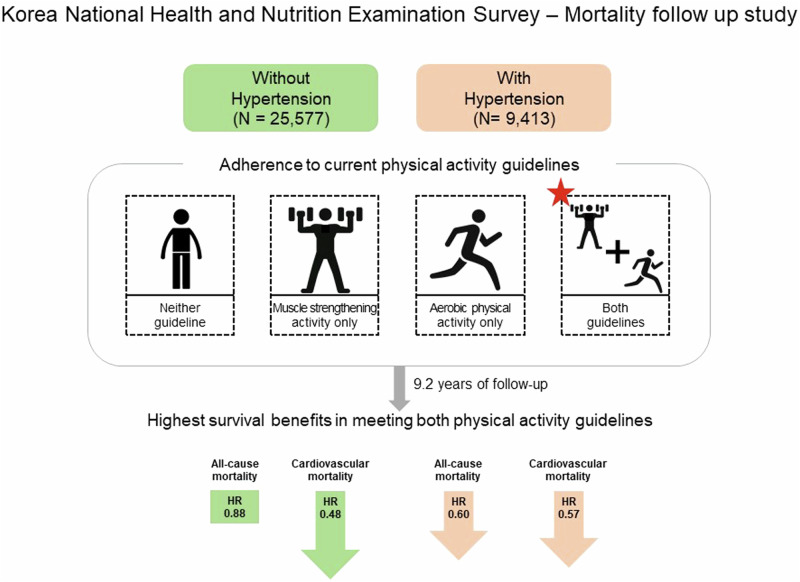

## Introduction

The benefits of physical activity (PA) in preventing various diseases and mortality are well established. Current PA guidelines for adults recommend at least 500 metabolic equivalent tasks (METs) (minutes per week) of aerobic PA and moderate- or high-intensity muscle-strengthening activity (MSA) that involves all major muscle groups on at least two days per week [1, 2]. Globally, 27.5% of adults do not meet the recommended level of aerobic PA [3], although meeting both aerobic PA and MSA guidelines remains low, with >80% of adults not meeting both recommendations [4]. However, the majority of existing studies on public health has focused only on the health benefits of aerobic PA, with less attention given to both components of the PA guidelines.

Hypertension is a major public health problem that substantially contributes to morbidity and mortality worldwide [5]. Physical activity recommendations for individuals with hypertension, including both aerobic PA and MSA guidelines, were similar to those for the general population [6, 7]. Although cumulative evidence has demonstrated the effectiveness of combining aerobic PA with MSA for managing blood pressure (BP) [8], studies on their impact on mortality remain relatively limited. Although some studies have found inverse dose-response associations for each aerobic PA [9–12] and MSA with mortality [13, 14] in hypertensive populations, the combined effects of engaging in both PA components on mortality remain poorly understood. Furthermore, it is unclear whether the relationships between the components of the PA guidelines and mortality vary between individuals with and without hypertension.

This study aimed to examine the associations of hypertension and meeting PA guidelines with the risk of all-cause and cardiovascular disease (CVD) mortalities.

Point of view

- Clinical relevance
  Among individuals with hypertension, meeting both aerobic physical activity and muscle strengthening activity guidelines is associated with greater benefits than engaging in either activity alone. Although hypertension is a leading cause of premature death, our findings indicate that adherence to minimal levels of aerobic physical activity and muscle strengthening guidelines may reduce some of this risk for individuals with hypertension.
- Future direction
  Our findings suggest that adherence to combined MSA and aerobic PA should be emphasized in individuals with hypertension. However, only engaging in MSA did not result in significant survival benefits. These findings highlight the need for further studies to identify the appropriate dosage of MSA for this population to maximize its health benefits.
- Consideration for the Asian population
  In Asian populations with hypertension, promoting the concurrent implementation of muscle strengthening and aerobic physical activity is important for optimizing health benefits and prevention of premature deaths.

## Methods

### Study population

**Fig. 1:**
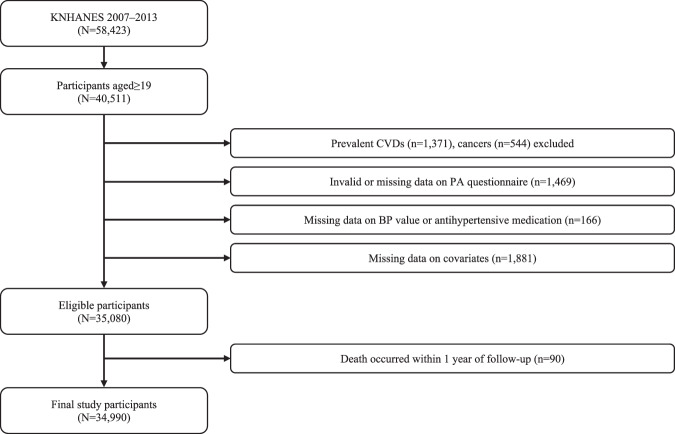
Flow diagram of eligible KNHANES participants

The KNHANES, conducted annually by the Korea Centers for Disease Control and Prevention (KCDC) since 1998, is a nationally representative survey that assesses the health and health-related behaviors of the Korean population. Further survey details are available in the literature [15]. The current analysis included adult KNHANES participants between 2007 and 2013 with available BP and PA measurements and mortality status assessed. Participants with self-reported cancers or CVDs, including angina pectoris, myocardial infarction, and stroke, were excluded from the study. To mitigate the potential effect of preclinical health conditions, participants who died within the first year were excluded (Fig. 1). Informed consent was obtained from all the participants before commencement of the study. The study procedures were approved by the KCDC and deemed IRB-exempt (IRB No. E2310/003-005) by the Seoul National University due to the analysis of publicly accessible, de-identified datasets.

### Hypertension

Trained medical staff conducted BP measurements using mercury sphygmomanometers and stethoscopes [15]. Following a 5-min resting period in the seated position, BP was measured three times with readings taken at 30-s intervals. The mean values of the second and third measurements were defined as systolic and diastolic BPs, respectively. In the 2008–2010 KNHANES, a review of the quality controls for BP measurements identified potential errors associated with the height of the participants’ arms. To address this concern, we adjusted the BP values from that period before incorporating them into our analysis. Hypertension was defined as systolic BP of ≥140 mmHg or diastolic BP of ≥90 mmHg, or self-reported use of antihypertensive medications [16].

### PA assessment

The study participants self-reported the frequency and duration of aerobic PA using the following questions adopted from the International Physical Activity Questionnaire [17]: (1) “Over the past week, on how many days did you engage in vigorous PA (e.g., running or jogging, mountain climbing, cycling at fast pace, fast swimming, and soccer) for ≥10 min that caused more exhaustion or larger increases in breathing than usual?; (2) “Over the past week, on how many days did you engage in moderate PA (e.g., slow swimming, doubles tennis, volleyball, badminton, and table tennis) for ≥10 min that caused slight exhaustion or a slightly increases in breathing than usual?; (3) “Over the past week, on how many days did you walk for ≥10 min?”. Participants who answered either question affirmatively were asked about the total duration of their participation in these activities, measured in hours and minutes. Metabolic equivalents of 8, 4, and 3.3 were assigned to vigorous PA, moderate PA, and walking, respectively [18]. Total amount of aerobic PA (METs-min/week) was calculated by summing the products of intensity, frequency, and duration of each activity. Additionally, MSA was assessed (days/week) using a self-reported response to the following question: “Over the past week, on how many days did you participate in muscle strengthening activity, such as push-up, sit-up, and weight lifting using dumbbells or barbells?” Current PA guidelines recommend that adults should engage in at least 500 METs-min/week of aerobic PA and 2 days/week of MSA [1, 2]. The participants were categorized into four groups based on adherence to guidelines on both aerobic PA and MSA [19]: (1) Neither (aerobic PA < 500 METs-min/week and MSA < 2 days/week); (2) Only MSA (aerobic PA < 500 METs-min/week and MSA ≥ 2 days/week); (3) Only aerobic PA (aerobic PA ≥ 500 METs-min/week and MSA < 2 days/week); and (4) Both (aerobic PA ≥ 500 METs-min/week and MSA ≥ 2 days/week).

### Mortality

The Korea Disease Control and Prevention Agency established a database that links KNHANES data to cause-of-death statistics provided by Statistics Korea. This linkage involves matching the KNHANES data with that of Cause of Death Statistics based on resident registration numbers for participants aged ≥19 years who consented to data linkage. The KNHANES data were combined with de-identified information on survival status until December 31, 2019 [20]. The causes of death were categorized according to the 10th version of the International Classification of Diseases [21]. We calculated both the total number of mortalities and specifically, mortalities related to CVD, coded as I00-I99.

### Covariates

Demographic and clinical information were collected at baseline in the survey. Based on existing literature, we selected covariates as follows: age (years), sex (male or female), household income (low, low-middle, middle-high, and high), educational level (≤elementary school, middle school, high school, and ≥ college), and marital status (unmarried, widowed or divorced, and married). Additionally, the participants self-reported lifestyle behaviors, such as smoking status (never smoked, former smoker, and present smoker) and alcohol consumption (non-heavy and heavy drinkers). Heavy drinkers were defined as having ≥7 drinks at least twice a week for males, and ≥5 drinks at least twice a week for females [22]. Body mass index (BMI) was calculated as measured weight (kg)/square of height (m^2^). Diabetes was defined as having a measured fasting glucose level of ≥126 mg/dL, self-reported use of antidiabetic medications, or a physician-diagnosed history of diabetes. Dyslipidemia was defined as having either a total cholesterol level of ≥240 mg/dL, low-density lipoprotein-cholesterol level of ≥160 mg/dL, high-density lipoprotein-cholesterol level of <40 mg/dL, triglyceride level of ≥200 mg/dL, or the use of lipid-lowering medication [23].

### Statistical analysis

Baseline characteristics were described according to the categories of hypertension status and the four levels of meeting the PA guidelines, with continuous variables reported as mean (standard deviation) and categorical variables as number (%). Cox proportional hazard models adjusted for potential confounders were used to estimate hazard ratios (HR) and 95% confidence intervals (CI) for the association of hypertension and PA with all-cause and CVD mortalities. The proportional hazards assumption was checked based on–the log plots. Models were constructed to estimate the association of hypertension and PA with mortality. Model 1 was adjusted for age and sex; Model 2 was adjusted for Model 1+ household income, educational level, marital status, smoking status, alcohol consumption, BMI, diabetes, and dyslipidemia; and Model 3 was additionally adjusted for both hypertension status and PA to assess independent associations with mortality risk. Subgroup analyses were performed to assess whether the relationship between adherence to the PA guidelines and mortality varied based on hypertension status. To assess the joint association of hypertension status and adherence to PA guidelines with mortality, the participants were cross-classified into eight categories based on hypertension status and four levels of adherence to the PA guidelines. Multiplicative interactions were assessed by comparing the two models, with and without an interaction term, using the log-likelihood ratio test. Sensitivity analyses were performed to minimize the potential risk of reverse causality by excluding mortalities within an additional period of two years. Furthermore, to investigate whether treatment status modifies the association between adherence to PA guidelines and mortality, we additionally conducted a stratified analysis by antihypertensive medication use among hypertensive individuals. All analyses were performed using R software (version 4.2.0). A two-tailed *P* < 0.05 was considered statistically significant.

## Results

### Baseline characteristics

Over a mean follow-up period of 9.2 years, 1948 deaths occurred, including 419 due to CVD. Baseline characteristics of 34,990 study participants were stratified by hypertension status and meeting the PA guidelines (Table 1). The average age of all the participants in the study was 48 years, with 20,102 of the 34,990 participants (57.5%) being female. Among the study participants, 27.0% had hypertension, tended to be older, more obese, and were more likely to have diabetes and dyslipidemia. Majority of participants engaged in only aerobic PA of ≥500 METs-min/week (52.6%), 2.4% engaged only in MSA for at least two days per week, 17.6% adhered to the complete guidelines, and 27.4% met neither of the guidelines.

**Table 1:** Baseline characteristics according to hypertension status and meeting PA guidelines

	Total (*N* = 34,990)	Hypertension status		Meeting PA guidelines			
Characteristics		Non-hypertension (*N* = 25,577)	Hypertension (*N* = 9413)	Neither (*N* = 9602)	MSA only (*N* = 841)	Aerobic PA only (*N* = 18,392)	Both (*N* = 6155)
Age, years (SD)	48.3 (15.9)	43.9 (14.7)	60.3 (12.8)	49.7 (16.5)	49.6 (14.8)	48.2 (15.9)	46.5 (15.0)
Female, *n* (%)	20,102 (57.5)	15,146 (59.2)	4956 (52.7)	6422 (66.9)	365 (43.4)	11,135 (60.5)	2180 (35.4)
BMI, kg/m^2^ (SD)	23.6 (3.4)	23.2 (3.2)	25.0 (3.4)	23.5 (3.5)	23.7 (3.0)	23.6 (3.4)	23.9 (3.1)
Systolic blood pressure, mmHg (SD)	117.4 (17.2)	110.8 (11.7)	135.5 (16.8)	117.7 (17.6)	118.3 (16.7)	117.3 (17.3)	117.5 (16.1)
Diastolic blood pressure, mmHg (SD)	75.1 (10.5)	72.4 (8.4)	82.5 (12.1)	74.6 (10.5)	76.0 (10.2)	75.0 (10.6)	76.2 (10.3)
Follow-up period, year (SD)	9.16 (2.03)	9.29 (1.97)	8.81 (2.16)	8.94 (2.08)	9.09 (1.95)	9.27 (2.02)	9.16 (1.97)
Cigarette smoking, *n* (%)							
Non-smoker	20,707 (59.2)	15,445 (60.4)	5262 (55.9)	6206 (64.6)	429 (51.0)	11,277 (61.3)	2795 (45.4)
Former smoker	6694 (19.1)	4331 (16.9)	2363 (25.1)	1506 (15.7)	197 (23.4)	3198 (17.4)	1793 (29.1)
Current smoker	7589 (21.7)	5801 (22.7)	1788 (19.0)	1890 (19.7)	215 (25.6)	3917 (21.3)	1567 (25.5)
Alcohol consumption, *n* (%)							
Non-heavy drinker	31,028 (88.7)	22,768 (89.0)	8260 (87.8)	8723 (90.8)	755 (89.8)	16,295 (88.6)	5255 (85.4)
Heavy drinker	3962 (11.3)	2809 (11.0)	1153 (12.2)	879 (9.2)	86 (10.2)	2097 (11.4)	900 (14.6)
Household income, *n* (%)							
Low	6492 (18.6)	3586 (14.0)	2906 (30.9)	2126 (22.1)	150 (17.8)	3454 (18.8)	762 (12.4)
Low-middle	8909 (25.5)	6442 (25.2)	2467 (26.2)	2,484 (25.9)	230 (27.3)	4736 (25.8)	1459 (23.7)
Middle-high	9642 (27.6)	7549 (29.5)	2093 (22.2)	2558 (26.6)	218 (25.9)	5103 (27.7)	1763 (28.6)
High	9947 (28.4)	8000 (31.3)	1947 (20.7)	2434 (25.3)	243 (28.9)	5099 (27.7)	2171 (35.3)
Educational level, *n* (%)							
≤Elementary school	8521 (24.4)	4210 (16.5)	4311 (45.8)	2934 (30.6)	167 (19.9)	4727 (25.7)	693 (11.3)
Middle school	3705 (10.6)	2384 (9.3)	1321 (14.0)	954 (9.9)	115 (13.7)	1937 (10.5)	699 (11.4)
High school	12,355 (35.3)	10,001 (39.1)	2354 (25.0)	2968 (30.9)	336 (40.0)	6426 (34.9)	2625 (42.6)
≥College	10,409 (29.7)	8982 (35.1)	1427 (15.2)	2746 (28.6)	223 (26.5)	5302 (28.8)	2138 (34.7)
Marital status, *n* (%)							
Unmarried	5177 (14.8)	4858 (19.0)	319 (3.4)	1093 (11.4)	107 (12.7)	2765 (15.0)	1212 (19.7)
Widowed or divorced	3973 (11.4)	1964 (7.7)	2009 (21.3)	1440 (15.1)	92 (10.9)	2058 (11.2)	383 (6.2)
Married	25,840 (73.8)	18,755 (73.3)	7085 (75.3)	7069 (73.6)	642 (76.3)	13,569 (73.8)	4560 (74.1)
Chronic conditions, *n* (%)							
Diabetes mellitus	3252 (9.3)	1376 (5.4)	1876 (19.9)	948 (9.9)	83 (9.9)	1689 (9.2)	532 (8.6)
Dyslipidemia	12,728 (36.4)	7814 (30.6)	4914 (52.2)	3654 (38.1)	323 (38.4)	6550 (35.6)	2201 (35.8)

*PA* physical activity, *BMI* body mass index, *MSA* muscle strengthening activity

### Association of hypertension and PA with all-cause and CVD mortalities

The associations between hypertension and PA with all-cause and CVD mortalities are summarized in Table 2. The interaction between exposure and sex was not significant (*P* > 0.05), and the associations were assessed by combining data from both males and females. Compared with non-hypertensive participants, hypertension was associated with higher risk of all-cause (HR, 1.11 [95% CI, 1.01–1.22]) and CVD (HR, 1.40 [95% CI, 1.14–1.73]) mortalities. For aerobic PA, compared with totally inactive individuals (0 METs-min/week), higher levels of aerobic PA (≥1000 METs-min/week) were associated with lower risk of all-cause (HR, 0.82 [95% CI, 0.72–0.93]) and CVD (HR, 0.67 [95% CI, 0.52–0.87]) mortalities. For MSA, 1 day/week (HR, 0.65 [95% CI, 0.47–0.90]) and 2–3 days/week (HR, 0.73 [95% CI, 0.59–0.89]) of engaging in MSA were associated with lower risk of all-cause mortality; however, these associations were not significant for CVD mortality. For participants meeting the PA guidelines, compared with those who met neither of the guidelines, meeting only the aerobic PA guideline had lower risk (HR, 0.86 [95% CI, 0.78–0.95] for all-cause mortality; HR, 0.78 [95% CI, 0.64–0.96] for CVD mortality). Moreover, those meeting both the guidelines had significantly lower risk of mortality (HR, 0.72 [95% CI, 0.61–0.85] for all-cause mortality; HR, 0.54 [95% CI, 0.36–0.80] for CVD mortality). Among all individuals with hypertension, 6008 participants (63.8%) were taking antihypertensive medication. There was no significant association between medication use and the risk of all-cause or CVD mortality (Supplementary Table 1).

**Table 2:** Independent associations of hypertension status and PA components with all-cause and CVD mortalities

Variables	No. of participants, *n*	No. of deaths, *n*	Hazard ratio (95% CI) for mortality		
			Model 1^a^	Model 2^b^	Model 3^c^
All-cause mortality					
*Hypertension status*					
Non-hypertension	25,577	909	1.00 (ref.)	1.00 (ref.)	1.00 (ref.)
Hypertension	9413	1038	1.03 (0.94, 1.13)	1.10 (1.00, 1.21)	1.11 (1.01, 1.22)
*Aerobic PA*					
0 METs–min/week	3628	351	1.00 (ref.)	1.00 (ref.)	1.00 (ref.)
1–499 METs–min/week	6815	371	0.85 (0.74, 0.99)	0.92 (0.80, 1.07)	0.93 (0.80, 1.08)
500–999 METs–min/week	5702	301	0.76 (0.65, 0.89)	0.85 (0.73, 1.00)	0.87 (0.74, 1.01)
≥1000 METs–min/week	18,845	924	0.72 (0.63, 0.81)	0.80 (0.70, 0.91)	0.82 (0.72, 0.93)
*MSA*					
0 day/week	25,885	1643	1.00 (ref.)	1.00 (ref.)	1.00 (ref.)
1 day/week	2109	39	0.57 (0.42, 0.79)	0.65 (0.47, 0.89)	0.65 (0.47, 0.90)
2–3 days/week	3976	103	0.62 (0.51, 0.76)	0.71 (0.58, 0.87)	0.73 (0.59, 0.89)
≥4 day/week	3020	162	0.75 (0.64, 0.89)	0.87 (0.74, 1.03)	0.90 (0.76, 1.06)
*Meeting PA guidelines*					
Neither	9602	679	1.00 (ref.)	1.00 (ref.)	1.00 (ref.)
MSA only	841	43	0.77 (0.56, 1.05)	0.85 (0.62, 1.16)	0.85 (0.62, 1.15)
Aerobic PA only	18,392	1003	0.82 (0.75, 0.91)	0.86 (0.78, 0.95)	0.86 (0.78, 0.95)
Both	6155	222	0.61 (0.52, 0.71)	0.72 (0.62, 0.85)	0.72 (0.61, 0.85)
CVD mortality					
*Hypertension status*					
Non-hypertension	25,577	155	1.00 (ref.)	1.00 (ref.)	1.00 (ref.)
Hypertension	9413	264	1.36 (1.11, 1.67)	1.39 (1.13, 1.72)	1.40 (1.14, 1.73)
*Aerobic PA*					
0 METs–min/week	3628	94	1.00 (ref.)	1.00 (ref.)	1.00 (ref.)
1–499 METs–min/week	6815	86	0.78 (0.59, 1.05)	0.85 (0.63, 1.14)	0.85 (0.64, 1.15)
500–999 METs–min/week	5702	66	0.67 (0.49, 0.92)	0.74 (0.54, 1.02)	0.75 (0.55, 1.03)
≥1000 METs–min/week	18,845	173	0.59 (0.46, 0.76)	0.65 (0.50, 0.85)	0.67 (0.52, 0.87)
*MSA*					
0 day/week	25,885	369	1.00 (ref.)	1.00 (ref.)	1.00 (ref.)
1 day/week	2109	5	0.43 (0.18, 1.04)	0.49 (0.20, 1.19)	0.50 (0.20, 1.21)
2–3 days/week	3976	19	0.65 (0.41, 1.05)	0.76 (0.48, 1.23)	0.80 (0.50, 1.30)
≥4 day/week	3020	26	0.64 (0.43, 0.96)	0.74 (0.49, 1.11)	0.78 (0.51, 1.18)
*Meeting PA guidelines*					
Neither	9602	167	1.00 (ref.)	1.00 (ref.)	1.00 (ref.)
MSA only	841	13	1.12 (0.63, 1.97)	1.24 (0.70, 2.19)	1.23 (0.69, 2.17)
Aerobic PA only	18,392	207	0.75 (0.61, 0.92)	0.78 (0.64, 0.96)	0.78 (0.64, 0.96)
Both	6155	32	0.46 (0.31, 0.68)	0.55 (0.37, 0.81)	0.54 (0.36, 0.80)

*CVD* cardiovascular disease, *PA* physical activity, *MSA* muscle strengthening activity, *METs* metabolic equivalent tasks

^a^Model 1: adjusted for age and sex

^b^Model 2: adjusted for Model 1 plus BMI (kg/m^2^), smoking status (non-smoker/former smoker/current smoker), alcohol consumption (non-heavy drinker/heavy drinker), household income (low/low-middle/middle-high/high), educational level (≤elementary school/middle school/high school/≥college), marital status (unmarried/widowed or divorced/married), diabetes (yes/no) and dyslipidemia (yes/no)

^c^Model 3: hypertension, aerobic PA, MSA, and meeting PA guidelines were further mutually adjusted as exposures in different analyses

### Stratified analyses and joint association

**Fig. 2:**
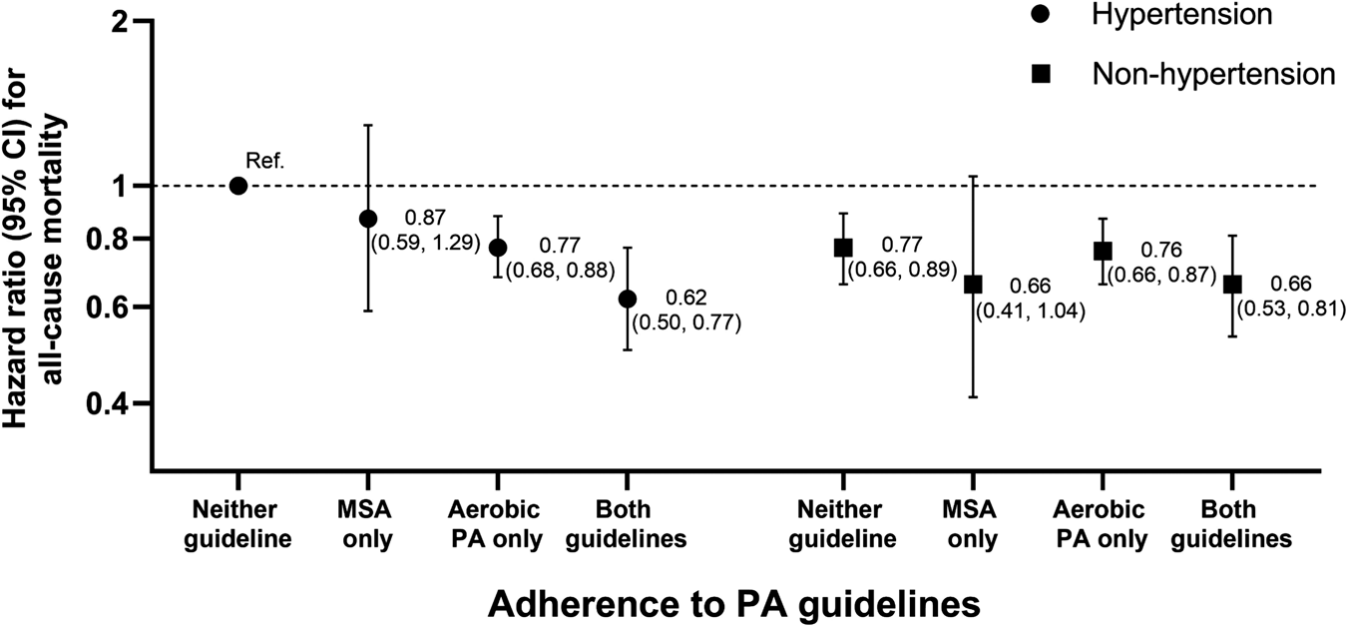
Hazard ratios of all-cause mortality by combinations of meeting PA guidelines and hypertension status. PA physical activity; and MSA muscle-strengthening activity. Adjusted for age, sex(male/female), BMI (kg/m^2^), smoking status (non-smoker/former smoker/current smoker), alcohol consumption (non-heavy drinker/heavy drinker), household income (low/low-middle/middle-high), educational level (≤elementary school/middle school/high school/≥college), marital status (unmarried/widowed or divorced/married), diabetes (yes/no) and dyslipidemia (yes/no)

**Fig. 3:**
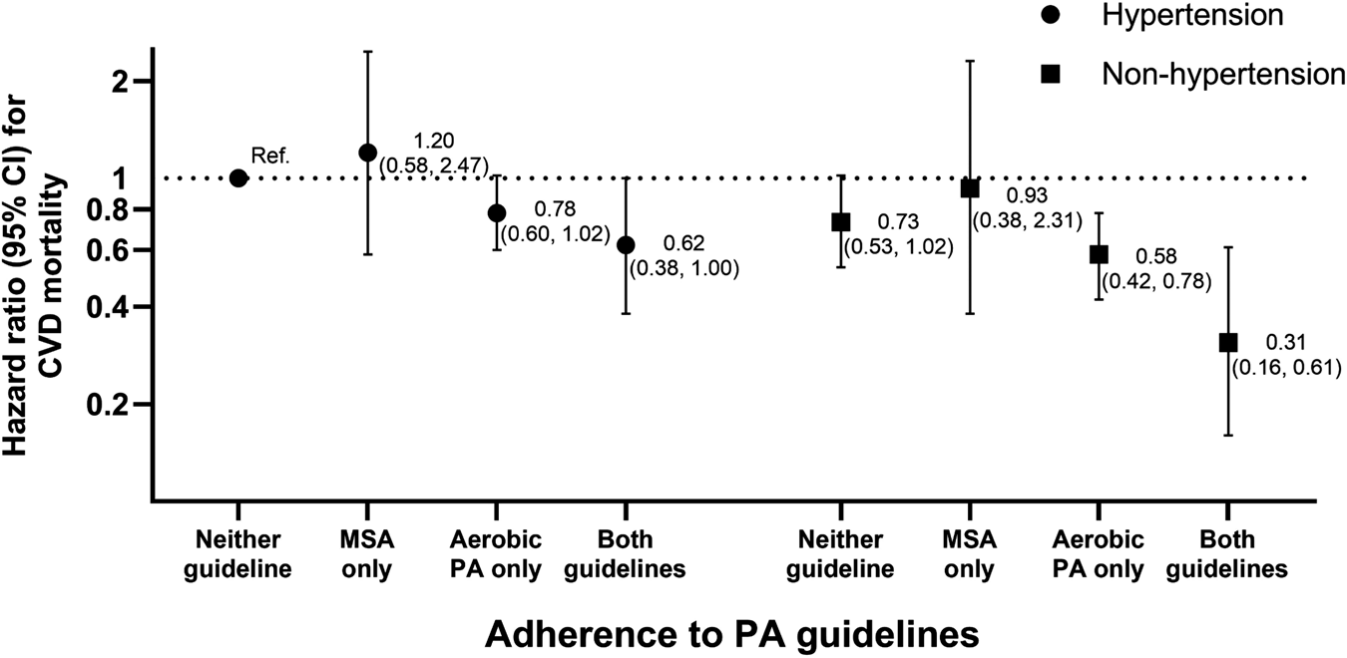
Hazard ratios of CVD mortality by combinations of meeting PA guidelines and hypertension status. CVD indicates cardiovascular diseases; PA physical activity; and MSA muscle-strengthening activity. Adjusted for age, sex(male/female), BMI (kg/m^2^), smoking status (non-smoker/former smoker/current smoker), alcohol consumption (non-heavy drinker/heavy drinker), household income (low/low-middle/middle-high), educational level (≤elementary school/middle school/high school/≥college), marital status (unmarried/widowed or divorced/married), diabetes (yes/no) and dyslipidemia (yes/no)

A significant interaction was observed between hypertension status and adherence to the PA guidelines for all-cause mortality (*P* = 0.02), but not for CVD mortality. Table 3 shows the stratified association of hypertension status and meeting PA guidelines with mortality. Among non-hypertensive participants, compared to those who met neither of the guidelines, there were no significant benefits of meeting any of PA guidelines for all-cause mortality. However, for CVD mortality, only the individuals who met both the guidelines was associated with lower risk (HR, 0.48 [95% CI, 0.24–0.97]). In contrast, the significant benefits of engaging in the recommended levels of PA were more evident among individuals with hypertension. Among individuals with hypertension, a substantial risk reduction for all-cause mortality and CVD mortality was observed in those who adhered to only the aerobic PA (HR, 0.76 [95% CI, 0.67–0.87] for all-cause mortality; HR, 0.76 [95% CI, 0.59–0.99] for CVD mortality) and both the PA guidelines (HR, 0.60 [95% CI, 0.48–0.76] for all-cause mortality; HR, 0.57 [95% CI, 0.35–0.92] for CVD mortality). However, there was no significant association between meeting only MSA guideline and risk of mortality. In sensitivity analyses, these results remained similar after excluding deaths during the first 3 years of follow-up (Supplementary Table 2), although there was no statistical significance regarding CVD mortality. Also, mortality risk reductions were observed in hypertensive individuals, regardless of antihypertensive medication use, when they met either the aerobic PA guideline or both guidelines (Supplementary Table 3). Figures 2 and 3 show the joint associations of hypertension and meeting the PA guidelines with mortalities. For all-cause mortality, compared to participants with hypertension meeting neither of the PA guidelines, the non-hypertensive participants had consistently lower risk except for those who adhered to only MSA guideline (HR, 0.66 [95% CI, 0.41–1.04]). Regarding CVD mortality, a significantly lower risk was observed in non-hypertensive participants who met either aerobic PA guideline alone (HR, 0.58 [95% CI, 0.42–0.78]) and both PA guidelines (HR, 0.31 [95% CI, 0.16–0.61]). Notably, among hypertensive participants, only those adhering to both PA guidelines had a significant benefit (HR, 0.62 [95% CI, 0.38–1.00], *P* = 0.05). There was no significant association between meeting only the MSA guideline and risk reduction on CVD mortality, consistent with the all-cause mortality findings. These results were similar even after excluding individuals who died during the first 3 years of follow-up (Supplementary Figs. 1, 2).

**Table 3:** Stratified associations of meeting PA guidelines with all-cause and CVD mortality within hypertension status

Variables	No. of participants, *n*	No. of deaths, *n*	Adjusted hazard ratio^a^ (95% CI) for mortality
All-cause mortality			
Non-hypertension group			
Meeting PA guidelines			
Neither	6871	268	1.00 (ref.)
MSA only	604	17	0.83 (0.50, 1.35)
Aerobic PA only	13,507	506	1.01 (0.87, 1.17)
Both	4595	118	0.88 (0.70, 1.11)
Hypertension group			
Meeting PA guidelines			
Neither	2731	411	1.00 (ref.)
MSA only	237	26	0.87 (0.59, 1.30)
Aerobic PA only	4885	497	0.76 (0.67, 0.87)
Both	1560	104	0.60 (0.48, 0.76)
CVD mortality			
Non-hypertension group			
Meeting PA guidelines			
Neither	6871	59	1.00 (ref.)
MSA only	604	5	1.36 (0.54, 3.40)
Aerobic PA only	13,507	81	0.80 (0.57, 1.12)
Both	4595	10	0.48 (0.24, 0.97)
Hypertension group			
Meeting PA guidelines			
Neither	2731	108	1.00 (ref.)
MSA only	237	8	1.11 (0.54, 2.30)
Aerobic PA only	4885	126	0.76 (0.59, 0.99)
Both	1560	22	0.57 (0.35, 0.92)

*CVD* cardiovascular disease, *PA* physical activity, *MSA* muscle strengthening activity

^a^Adjusted for age, sex(male/female), BMI (kg/m^2^), smoking status (non-smoker/former smoker/current smoker), alcohol consumption (non-heavy drinker/heavy drinker), household income (low/low-middle/middle-high), educational level (≤elementary school/middle school/high school/≥college), marital status (unmarried/widowed or divorced/married), diabetes (yes/no) and dyslipidemia (yes/no)

## Discussion

This study examined the comprehensive association of hypertension status and PA level with all-cause and CVD mortality in the general population. Overall, the participants with hypertension were consistently associated with a higher risk of all-cause and CVD mortalities, independent of PA. These results support previous findings [24, 25]. Park et al. [25] observed a higher risk of all-cause mortality related to hypertension in two population-based cohorts. In contrast, the increase in all-cause mortality risk (19–28%) associated with hypertension among Koreans, as reported in their study, was higher than that observed in our study (11%). This discrepancy could be attributed to differences in the age range of the study participants (20–80 vs. 40–69) and confounding factors. Park et al. also reported that engaging in moderate-to-vigorous PA at least once a week was associated with a lower mortality risk. However, as the level of PA was defined only by frequency and MSA was not considered, it is not feasible to directly compare the benefits of the recommended PA levels on mortality.

A recent meta-analysis [26] reported that MSA is associated with a lower risk of mortality, including death due to CVD. In our study, the participants engaged in MSA 1–3 days/week had a 27–38% reduction in all-cause mortality after adjusting for potential confounders, including the aerobic PA; however, this association was not shown in CVD mortality. Our findings are consistent with those of Stamatakis et al. [27] who observed a 23% reduction in all-cause mortality without a statistically significant change in CVD mortality. However, given the relatively small sample size and possibility that CVD-related deaths may have influenced these results [28], further investigation with larger sample sizes are warranted to confirm these findings. Additionally, the present study found that meeting the guidelines for both aerobic PA and MSA was associated with a significantly lower risk of mortality in the total study sample. Similar to previous studies in the general population [29, 30], our findings support the current PA guidelines and suggest that incorporating MSA in combination with aerobic PA may provide a greater survival benefit. It has been well established that aerobic PA and MSA provide various health benefits [31], including cardiometabolic risk factors [32–34], increased strength [35] and fitness [36], reduced visceral fat [37], and mitigation of depressive symptoms [38], all contributing to a lower risk of mortality.

In the subgroup analysis, the protective effect of engaging in a minimal level of the recommended aerobic PA guidelines and both guidelines, including MSA, was more prominent in individuals with hypertension. These results support the previous findings that individuals with CVD benefit more from PA than those without CVD [39]. Potential mechanisms underlying the additional benefit of exercise in individuals with hypertension may include improvements in endothelial function [40] and other cardiovascular risk [41], particularly in achieving a greater reduction in BP compared to non-hypertensive individuals [8, 42], thereby leading to subsequent reductions in mortality risk. To our knowledge, no comparable study has examined the association between meeting the PA guidelines and mortality risk associated with hypertension. Our joint analysis revealed that although a non-hypertensive status was associated with a greater reduction in CVD mortality risk, meeting both the PA guidelines may attenuate the detrimental effects of hypertension on CVD mortality. Notably, individuals with hypertension who met both the PA guidelines showed a relatively lower mortality risk than inactive normotensive individuals. These findings support the prioritization of promoting combined aerobic PA and MSA to reduce the risk of premature deaths among hypertensive populations in a public health context.

However, our findings regarding meeting only the MSA guidelines suggest that engaging in MSA without performing the recommended level of aerobic PA may not necessarily offer additional health benefits, especially for CVD mortality. The HR estimates for CVD mortality, although not statistically significant, suggest a potential adverse effect of MSA on CVD mortality. Similarly, previous studies have observed no significant differences in CVD mortality between individuals meeting only the MSA guidelines and those not meeting either guideline [27, 43]. Furthermore, studies found a potential quadratic association between MSA and CVD risks, with individuals engaged in the highest amount of weight training (>5 h/week) exhibiting elevated CVD risk (HR, 1.24 [95% CI, 0.73–2.09]) after adjusting the aerobic PA [44]. Similarly, those engaged in the highest frequency of resistance training (≥4 times/week) showed the highest CVD risk (HR, 1.33 [95% CI, 0.75–2.36]) [45]. Possible explanations for this observation could be arterial stiffness and a potential mild form of cardiac hypertrophy related to a high volume of resistance training [46, 47], subsequently leading to both all-cause and CVD mortalities [48]. However, the relationship between MSA levels and CVD mortality remains unclear. Evidence from meta-analyses suggests that low to moderate-intensity resistance training [49] and engaging in resistance training twice a week [50] are associated with improvement in arterial stiffness. Further studies are needed to determine the optimal intensity and duration of MSA therapy for CVD mortality.

This study has several limitations. First, self-reported aerobic PA and MSA may be susceptible to recall bias and misclassification compared to data obtained using objective measurements such as accelerometers and grip strength dynamometers. Second, the assessment of MSA was based only on frequency, without information on intensity or duration. Third, limited statistical power may have affected certain results, particularly within the group that met only the MSA guidelines. Fourth, owing to the cross-sectional nature of the survey, changes in PA behaviors over time could not be determined. Fifthly, hypertension status was solely categorized based on in-office BP measurements, which may have resulted in misclassification due to the potential presence of masked hypertension and white-coat hypertension. Finally, as an observational study, residual and unknown confounders could not be fully eliminated, thereby limiting the strength of the causal associations. Therefore, the conclusions regarding causality should be interpreted with caution.

### Asian perspectives

In the context of hypertension management, our findings underscore the significance of combined exercise as a key strategy for preventing premature deaths in individuals with hypertension. Furthermore, this study strengthens the evidence supporting the additional benefits of engaging in MSA along with aerobic PA in this population. However, low adherence to both PA guidelines was observed across the study population, with adherence rates below 20% (15.0% in Europe [51], 16.0% in the United States [19], and 17.6% in this Korean sample). Similarly, among individuals with hypertension in this study, only 16.6% met both guidelines. This low compliance underscores the critical importance of advocating for concurrent participation in both aerobic PA and MSA as a public health strategy. Such promotion is essential for attenuating the risks associated with various morbidities [52] and mortality.

## Conclusions

In this study, we found that meeting both PA guidelines was associated with greater survival benefits, regardless of hypertension status. Additionally, the advantage of meeting both PA guidelines was more evident among individuals with hypertension. While hypertension remains a significant risk factor for mortality, our study suggests that engaging in both recommended PA components, even at the minimum level, may mitigate some of this risk for individuals with hypertension. These findings support the importance of promoting MSA along with aerobic PA in public health efforts, especially for reducing the risk of premature deaths among individuals with hypertension.

## Supplementary Information

Supplementary Information

## Data Availability

All data were obtained from the Korea National Health and Nutrition Examination Survey (KNHANES, https://knhanes.kdca.go.kr/knhanes/main.do), following the officially documented data access procedures.
