# Supplementary material for: Combined association of aerobic and muscle strengthening activity with mortality in individuals with hypertension
Source: Hypertens Res. 2024 Aug 13;47(11):3056–67. doi: 10.1038/s41440-024-01788-3 (PMC11534690; doi:10.1038/s41440-024-01788-3)
Supplement: Supplementary file 1 — Supplementary Information [file 41440_2024_1788_MOESM1_ESM.docx]

**Association Combined association of aerobic and muscle strengthening activity with mortality in individuals with hypertension**

**Running Title:** Physical activity, hypertension, and mortality

Younghwan Choi, Duch-cul Lee, Yunmin Han, Hoyong Sung, Jiyeon Yoon, Yeon Soo Kim*

From the Department of Physical Education, Seoul National University, Seoul, South Korea (Y. C, Y. H); Department of Kinesiology, Iowa State University, Ames, IA 50011, USA (D-C. L, J. Y); Department of Physical Education, Korea Military Academy, Seoul, South Korea (H. S); Department of Physical Education, College of Education, Seoul National University, Seoul, South Korea; Institute of Sport Science, Seoul National University, Seoul, South Korea (Y.S, K)

***Corresponding author**

Yeon Soo Kim

Department of Physical Education

Seoul National University

1 Gwanak-ro, Gwanak-gu, Seoul

South Korea, 08826

Tel: +82 02 880 7784

E-mail: kys0101@snu.ac.kr

**Supplementary Materials**

| **Supplementary Table 1. Association between antihypertension medication use and all-cause and CVD mortalities among hypertensive individuals.**  **Supplementary Table 2. Stratified associations of meeting PA guidelines with all-cause and CVD mortality within hypertension status, excluding deaths that occurred within 3 years of follow-up.**  **Table S3. Stratified associations of meeting PA guidelines with all-cause and CVD mortality within treatment status, among hypertensive individuals.**  **Supplementary Figure 1.** **Hazard ratios of all-cause mortality by combinations of meeting PA guidelines and hypertension status, excluding deaths that occurred within 3 years of follow-up.**  **Supplementary Figure 2.** **Hazard ratios of CVD mortality by combinations of meeting PA guidelines and hypertension status, excluding deaths that occurred within 3 years of follow-up.** |
| --- |
|  |

**Supplementary Table 1. Association between antihypertension medication use and all-cause and CVD mortalities among hypertensive individuals.**

| **Variables** | **No. of participants, *n*** | **No. of deaths, *n*** | **Adjusted hazard ratio* (95% CI) for mortality** |
| --- | --- | --- | --- |
| All-cause mortality |  |  |  |
| Antihypertensive medication use |  |  |  |
| No | 3,405 | 279 | 1.00 (ref.) |
| Yes | 6,008 | 759 | 0.91 (0.78, 1.05) |
| CVD mortality |  |  |  |
| Antihypertensive medication use |  |  |  |
| No | 3,405 | 59 | 1.00 (ref.) |
| Yes | 6,008 | 759 | 1.12 (0.82, 1.51) |

CVD indicates cardiovascular disease

* Adjusted for age, sex(male/female), BMI (kg/m^2^), smoking status (non-smoker/former smoker/current smoker), alcohol consumption (non-heavy drinker/heavy drinker), household income (low/low-middle/middle-high), educational level (≤elementary school/middle school/high school/≥college), marital status (unmarried/widowed or divorced/married), diabetes (yes/no), dyslipidemia (yes/no), and meeting PA guidelines (neither/aerobic PA only/MSA only/both).

**Supplementary Table 2. Stratified associations of meeting PA guidelines with all-cause and CVD mortality within hypertension status, excluding deaths that occurred within 3 years of follow-up.**

| **Variables** | **No. of participants, *n*** | **No. of deaths, *n*** | **Adjusted hazard ratio* (95% CI) for mortality** |
| --- | --- | --- | --- |
| All-cause mortality |  |  |  |
| Non-hypertension group |  |  |  |
| Meeting PA guidelines |  |  |  |
| Neither | 6,817 | 214 | 1.00 (ref.) |
| Muscle only | 603 | 16 | 0.99 (0.60, 1.65) |
| Aerobic only | 13,435 | 434 | 1.08 (0.92, 1.27) |
| Both | 4,574 | 97 | 0.93 (0.72, 1.19) |
| Hypertension group |  |  |  |
| Meeting PA guidelines |  |  |  |
| Neither | 2,668 | 348 | 1.00 (ref.) |
| Muscle only | 236 | 25 | 1.00 (0.67, 1.51) |
| Aerobic only | 4,806 | 418 | 0.74 (0.64, 0.86) |
| Both | 1,542 | 86 | 0.58 (0.45, 0.75) |
| CVD mortality |  |  |  |
| Non-hypertension group |  |  |  |
| Meeting PA guidelines |  |  |  |
| Neither | 6,817 | 45 | 1.00 (ref.) |
| Muscle only | 603 | 5 | 1.86 (0.73, 4.73) |
| Aerobic only | 13,435 | 72 | 0.94 (0.64, 1.37) |
| Both | 4,574 | 9 | 0.62 (0.29, 1.30) |
| Hypertension group |  |  |  |
| Meeting PA guidelines |  |  |  |
| Neither | 2,668 | 85 | 1.00 (ref.) |
| Muscle only | 236 | 8 | 1.40 (0.67, 2.92) |
| Aerobic only | 4,806 | 104 | 0.78 (0.58, 1.04) |
| Both | 1,542 | 20 | 0.62 (0.37, 1.05) |

CVD indicates cardiovascular disease; PA, physical activity; and MSA, muscle strengthening activity

* Adjusted for age, sex(male/female), BMI (kg/m^2^), smoking status (non-smoker/former smoker/current smoker), alcohol consumption (non-heavy drinker/heavy drinker), household income (low/low-middle/middle-high), educational level (≤elementary school/middle school/high school/≥college), marital status (unmarried/widowed or divorced/married), diabetes (yes/no) and dyslipidemia (yes/no)

**Table S3. Stratified associations of meeting PA guidelines with all-cause and CVD mortality within treatment status, among hypertensive individuals.**

| **Variables** | **No. of participants, *n*** | **No. of deaths, *n*** | **Adjusted hazard ratio* (95% CI) for mortality** |
| --- | --- | --- | --- |
| All-cause mortality |  |  |  |
| Antihypertensive medication non-user |  |  |  |
| Meeting PA guidelines |  |  |  |
| Neither | 890 | 110 | 1.00 (ref.) |
| Muscle only | 94 | 7 | 0.55 (0.25, 1.20) |
| Aerobic only | 1,802 | 131 | 0.68 (0.52, 0.88) |
| Both | 619 | 31 | 0.58 (0.39, 0.90) |
| Antihypertensive medication user |  |  |  |
| Meeting PA guidelines |  |  |  |
| Neither | 1,841 | 301 | 1.00 (ref.) |
| Muscle only | 143 | 19 | 1.03 (0.64, 1.64) |
| Aerobic only | 3,083 | 366 | 0.81 (0.69, 0.94) |
| Both | 941 | 73 | 0.62 (0.47, 0.82) |
| CVD mortality |  |  |  |
| Antihypertensive medication non-user |  |  |  |
| Meeting PA guidelines |  |  |  |
| Neither | 890 | 21 | 1.00 (ref.) |
| Muscle only | 94 | 2 | 0.93 (0.21, 4.10) |
| Aerobic only | 1,802 | 31 | 0.86 (0.49, 1.52) |
| Both | 619 | 5 | 0.54 (0.19, 1.48) |
| Antihypertensive medication user |  |  |  |
| Meeting PA guidelines |  |  |  |
| Neither | 1,841 | 87 | 1.00 (ref.) |
| Muscle only | 143 | 6 | 1.22 (0.53, 2.81) |
| Aerobic only | 3,083 | 95 | 0.76 (0.57, 1.03) |
| Both | 941 | 17 | 0.60 (0.34, 1.04) |

CVD indicates cardiovascular disease; PA, physical activity; and MSA, muscle strengthening activity

* Adjusted for age, sex(male/female), BMI (kg/m^2^), smoking status (non-smoker/former smoker/current smoker), alcohol consumption (non-heavy drinker/heavy drinker), household income (low/low-middle/middle-high), educational level (≤elementary school/middle school/high school/≥college), marital status (unmarried/widowed or divorced/married), diabetes (yes/no) and dyslipidemia (yes/no)

**Supplementary Figure 1.** **Hazard ratios of all-cause mortality by combinations of meeting PA guidelines and hypertension status, excluding deaths that occurred within 3 years of follow-up.**


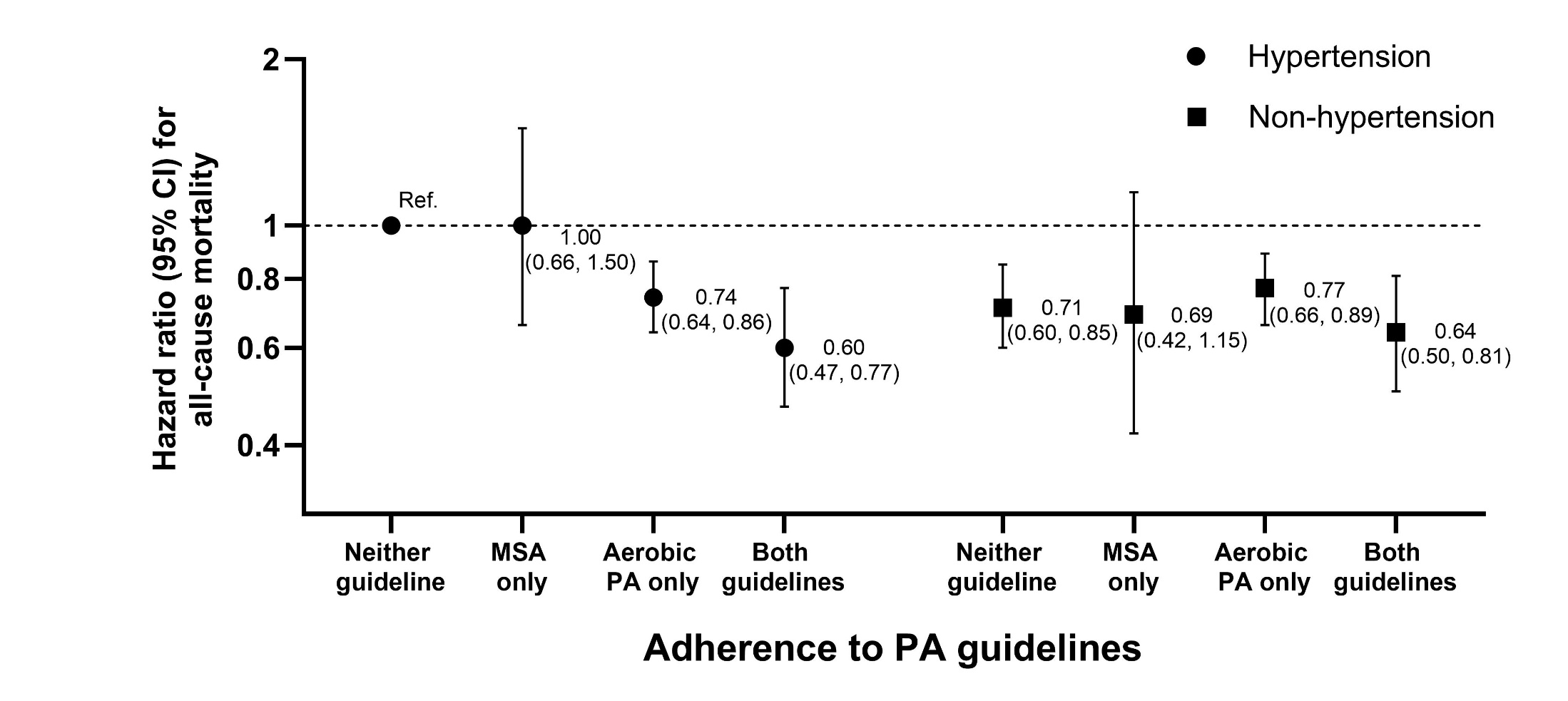


PA, physical activity; and MSA, muscle strengthening activity

Adjusted for age, sex(male/female), BMI (kg/m^2^), smoking status (non-smoker/former smoker/current smoker), alcohol consumption (non-heavy drinker/heavy drinker), household income (low/low-middle/middle-high), educational level (≤elementary school/middle school/high school/≥college), marital status (unmarried/widowed or divorced/married), diabetes (yes/no) and dyslipidemia (yes/no)

**Supplementary Figure 2.** **Hazard ratios of CVD mortality by combinations of meeting PA guidelines and hypertension status, excluding deaths that occurred within 3 years of follow-up.**


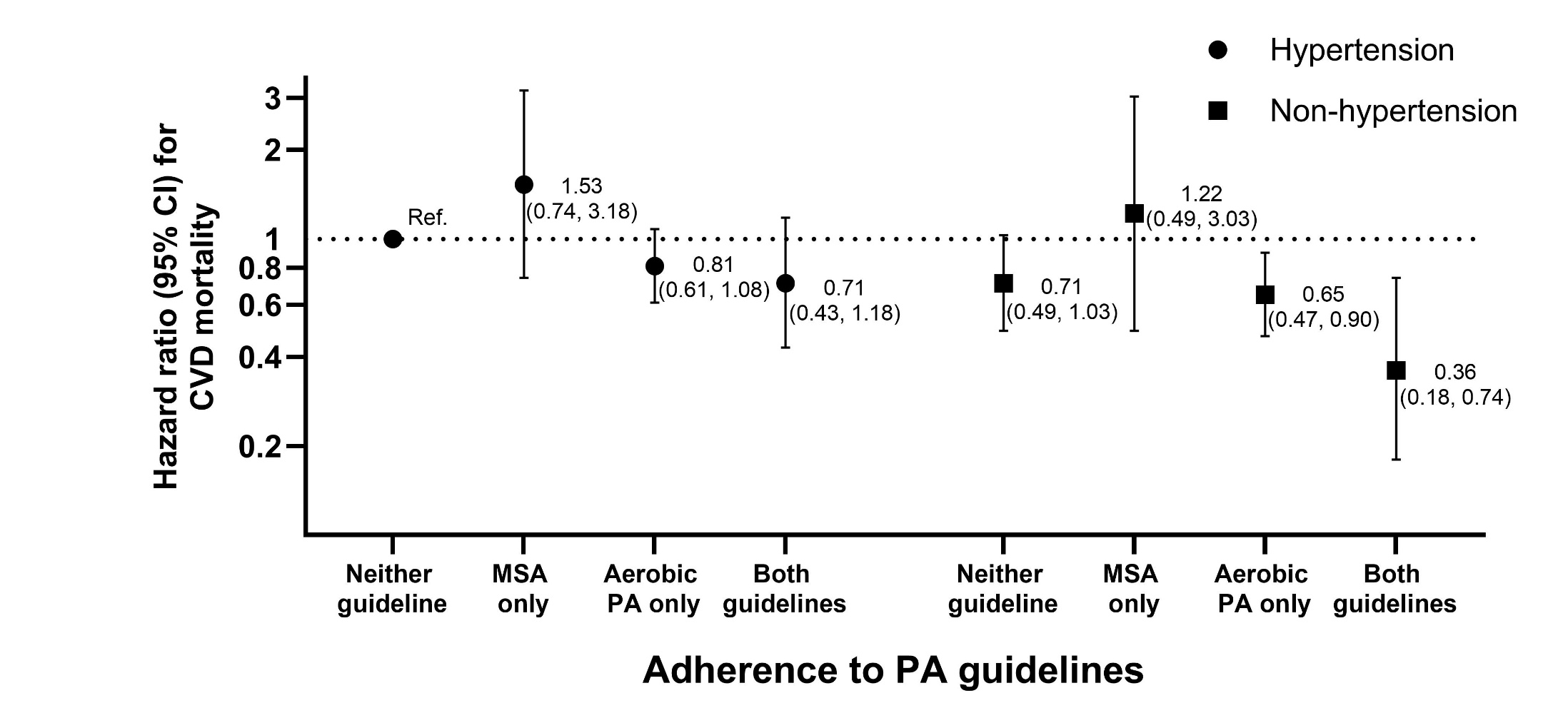


CVD indicates cardiovascular disease; PA, physical activity; and MSA, muscle strengthening activity

Adjusted for age, sex(male/female), BMI (kg/m^2^), smoking status (non-smoker/former smoker/current smoker), alcohol consumption (non-heavy drinker/heavy drinker), household income (low/low-middle/middle-high), educational level (≤elementary school/middle school/high school/≥college), marital status (unmarried/widowed or divorced/married), diabetes (yes/no) and dyslipidemia (yes/no)
